# COVID-19: Multisystem Inflammatory Syndrome in Children (MIS-C)

**DOI:** 10.7759/cureus.21064

**Published:** 2022-01-09

**Authors:** Abdullah Aldawas, Mateen Ishfaq

**Affiliations:** 1 Pediatrics, King Saud Hospital, Unayzah, SAU

**Keywords:** toxic-shock syndrome, child health, kawasaki disease, multisystem inflammatory syndrome in children (mis-c), covid-19, coronavirus

## Abstract

A novel coronavirus was identified in late 2019 that rapidly reached pandemic proportions. The World Health Organization has designated the disease as COVID-19, which stands for coronavirus disease 2019. In children, COVID-19 is usually mild. However, in rare cases, children can be severely affected, and clinical manifestations may differ from adults. In April of 2020, reports from the United Kingdom documented a presentation in children similar to incomplete Kawasaki disease (KD) or toxic shock syndrome. Since then, there have been reports of similarly affected children in other parts of the world. The condition has been termed multisystem inflammatory syndrome in children (MIS-C). We report a case of a 12-year-old previously healthy boy admitted with fever, generalized skin rash, conjunctivitis, and multiorgan dysfunction with positive COVID-19 polymerase chain reaction (PCR), and diagnosed as MIS-C on the basis of clinical and laboratory criteria. He received intravenous immunoglobulin (IVIG) for two days and other supportive treatment. He improved with defervescence and normalization of acute-phase reactants.

## Introduction

Children with coronavirus disease 2019 (COVID-19) usually present with relatively mild symptoms. In the Spring of 2020, case reports emerged from the United Kingdom and neighboring countries which described an illness like Kawasaki disease (KD) in children and adolescents related to severe acute respiratory syndrome coronavirus 2 (SARS-CoV-2) infection. This condition later was labeled as multisystem inflammatory syndrome in children (MIS-C). MIS is considered a rare but serious complication of SARS-CoV-2 infection. The exact pathophysiology and incidence of MIS remain to be determined.

The pathophysiology of MIS-C is not well understood. It is thought to be a result of an altered immune response to the virus, with quite a few clinical similarities to KD, macrophage activation syndrome (MAS), and cytokine release syndrome. However, MIS-C is noted to have an immunological phenotype which is distinct from KD and MAS. Most affected children showed a positive serology for SARS-CoV-2 with negative polymerase chain reaction (PCR), a finding that further supports the hypothesis that MIS-C is related to immune dysregulation occurring after acute infection has passed. However, some children do have a positive PCR test result. In the early case series, 783 children were enrolled in whom both PCR and serology were performed [1-5]. Out of these, 60% showed positive serology with negative PCR, 34% turned out to be positive on both tests, and 5% had negative serology as well as PCR [6-8].

The clinical symptoms of MIS-C usually include continuous fevers, gastrointestinal symptoms (abdominal pain, vomiting, diarrhea), skin rashes, and conjunctivitis. Patients typically present with three to five days of fever, associated later with development of shock and/or multisystem involvement. Laboratory findings include lymphopenia, high inflammatory markers (C-reactive protein [CRP], erythrocyte sedimentation rate [ESR], D-dimers), and elevated cardiac markers (troponin, brain natriuretic peptide [BNP]). MIS-C may occur at any age from infancy through late adolescence. Most of cases with the illness are previously healthy children between the ages of 6 to 12 years. Black and Hispanic children appear to be disproportionally affected. Peaks of MIS-C cases have been seen several weeks after surges of COVID-19 in the community [8-11].

Differential diagnosis of MIS-C should include KD not related to SARS-CoV-2, bacterial sepsis, severe acute COVID-19, toxic shock syndrome, appendicitis and other viral infections like Epstein bar virus, adenovirus, cytomegalovirus and enteroviruses [1,3]. Children with severe MIS-C who present with a cytokine storm like picture are believed to be linked to delayed interferon gamma responses and slow clearance of viral load, further leading to heightened inflammatory reactions [7,8]. They present with systemic signs of inflammation, often with significant cardiac, neurologic, and/or hematologic dysfunction. They require a multidisciplinary team of healthcare providers including intensivists, pediatric infectious disease, rheumatology, cardiology and hematology specialists. Various guidelines for management have been proposed by several specialty societies.

Management of MIS-C has and is still evolving over the course of the pandemic. Most children with moderate-to-severe manifestations have been treated initially with both intravenous immunoglobulin (IVIG) and glucocorticoids. Additional therapies depend upon the severity and response to initial therapy. The benefit to risk ratio of adjunctive therapies (interleukin [IL] 1 inhibitors [eg, anakinra, canakinumab], IL-6 inhibitors [eg, tocilizumab]) are still uncertain. Consultations from pediatric infectious disease and rheumatology specialists should be taken.

The prognosis of MIS-C requires further description but overall looks positive as most children showed complete recovery. Long-term follow-up studies are limited. The disease course in MIS-C can be quite severe, with many children requiring intensive care admission. A big percentage of children have survived with occasional mortality [11,12].

## Case presentation

A 10-year-old Saudi boy presented to the emergency department (A&E) of King Saud Hospital with complaints of fever, skin rash, and conjunctivitis of two days’ duration. He was well two days ago when he developed fever, which was intermittent initially and later became continuous; the fever was recorded up till 40 °C, relieved with antipyretic (acetaminophen) for a few hours before returning with rigors and chills. It was associated with generalized malaise and body aches. Skin rash appeared within 48 hours of onset of fever, on the face and then descended on the trunk and limbs. It was nonpruritic and without any skin bleed and was associated with mild swelling of hands, feet, and lips. He also developed redness of eyes but without watering, stickiness, or any pain on movement of eyeballs. There was a history of abdominal pain in the epigastrium and right hypochondrium with few grade III non-bloody stools. There was no associated history of photophobia, seizures, vomiting, pain abdomen, palpitations, breathing difficulty, joint pains or swelling, and urinary complaints or contact with any person with a similar illness at home. About four weeks ago, all family was diagnosed with PCR-positive COVID-19 infection after one of the parents developed symptoms of fever and body ache. No other family member developed any symptoms but they completed their isolation for 10 days. Repeat testing was not done.

The patient was otherwise a healthy child who was fully vaccinated and nutritionally well built. He was admitted to the pediatric medical ward (in an isolation room) with a provisional diagnosis of nonspecific viral exanthema. His initial blood workup and COVID PCR were taken and he was started on antipyretics and intravenous fluids to address fever and hydration. On examination, the patient had toxic look with a heart rate (HR) of 123/min, respiratory rate of 30/min, temperature of 40 °C, and blood pressure of 90/58 mmHg. His oxygen saturation was maintained above 94% at room air. He was mildly dehydrated and there was generalized maculopapular rash involving the face and body with mild non-pitting edema of hands and feet. Lips were swollen, red with congested oral mucosa and tonsils. There were no ulcerations or vesicles in the oral cavity and pharyngeal wall. He had non-exudative conjunctivitis with normal movement of eyeballs. His cervical lymph nodes were enlarged significantly bilaterally. There was no evidence of skin bleeds. He had mild tenderness in right hypochondrium and his liver was palpable with a total span of 12 cm; other organs were not palpable and no clinical evidence of free fluid was found in abdominal cavity. His Glasgow Coma Scale was 15/15 with no neurological deficit and signs of meningeal irritation. His chest was clear and cardiac examination was unremarkable. His initial investigations revealed a white blood cell count (WBC) of 8,400/ul, hemoglobin (Hb) of 14g/dl, and platelet count of 133000/ul. His CRP turned out to be 6 mg/dl. He had mild elevation of liver enzymes - alanine aminotransferase (ALT) 50 IU/L and aspartate aminotransferase (AST) 54 IU/L but normal serum electrolytes and renal functions. He was kept under observation in isolation room. His COVID-19 PCR returned positive within 24 hours.

After complete examination and investigations, a provisional diagnosis of Kawasaki-like illness vs MIS-C was suspected. Pediatric rheumatologist and infectious disease were consulted; they advised a second panel of investigations: ESR, CRP, albumin, throat swab, antistreptolysin O (ASO) titer, serum ferritin, lactate dehydrogenase (LDH) albumin, D dimers, fibrin degradation product (FDP), prothrombin time (PT), activated partial thromboplastin time (APTT). His ESR was 22mm/h, CRP increased to 24 mg/dl, albumin: 24 g/L, ferritin: 886 ng/ml, LDH: 316 U/L, PT/APTT, coagulations studies and ASO titer were in normal range. Throat swab revealed normal flora. With 48 hours of admission, he started complaining of palpitations and mild chest pain and his blood pressure dropped to 5th centile for his height and age without any fluid loss. As his clinical features and laboratory values fulfilled the criteria for MIS-C (American college of Rheumatology) with a link to COVID 19, treatment was planned.

He was given crystalloid fluid boluses to treat hypotension and IVIG was started at 1 gm/kg/day for two days. His echocardiography was done which did not show any evidence of coronary artery aneurysm with good cardiac function. He was also given empirical antibiotic (ceftriaxone) and other supportive treatment. He showed dramatic response to IVIG and became afebrile within 24 hours of starting IVIG. His acute-phase reactants repeated after 48 hours also decreased considerably - ESR: 12 mm/h, CRP: 12 mg/dl, LDH: 200 and ferritin 500 ng/ml). He remained admitted for 10 days and did not show signs of deterioration. His blood culture showed no growth for any microorganism. He was discharged in good condition with follow up of pediatric cardiology within 14 days for a repeat echocardiogram. He was followed after two weeks in outpatient department. His echocardiogram was normal and his acute-phase reactants dropped to normal range. He was active, well hydrated and resumed his routine activities without any complaint.

**Figure 1:**
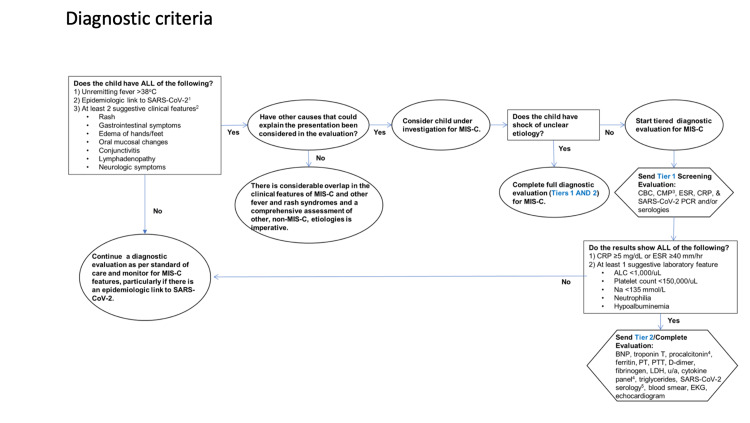
Diagnostic Pathway for MIS-C SARS-CoV-2: severe acute respiratory syndrome coronavirus 2; MIS-C: multisystem inflammatory syndrome in children; CBC: complete blood count; CMP: comprehensive metabolic panel; ESR: erythrocyte sedimentation rate; CRP: C-reactive protein; PCR: polymerase chain reaction; ALC: absolute lymphocyte count; PT: prothrombin time; PTT: partial thromboplastin time; LDH: lactate dehydrogenase.

**Figure 2:**
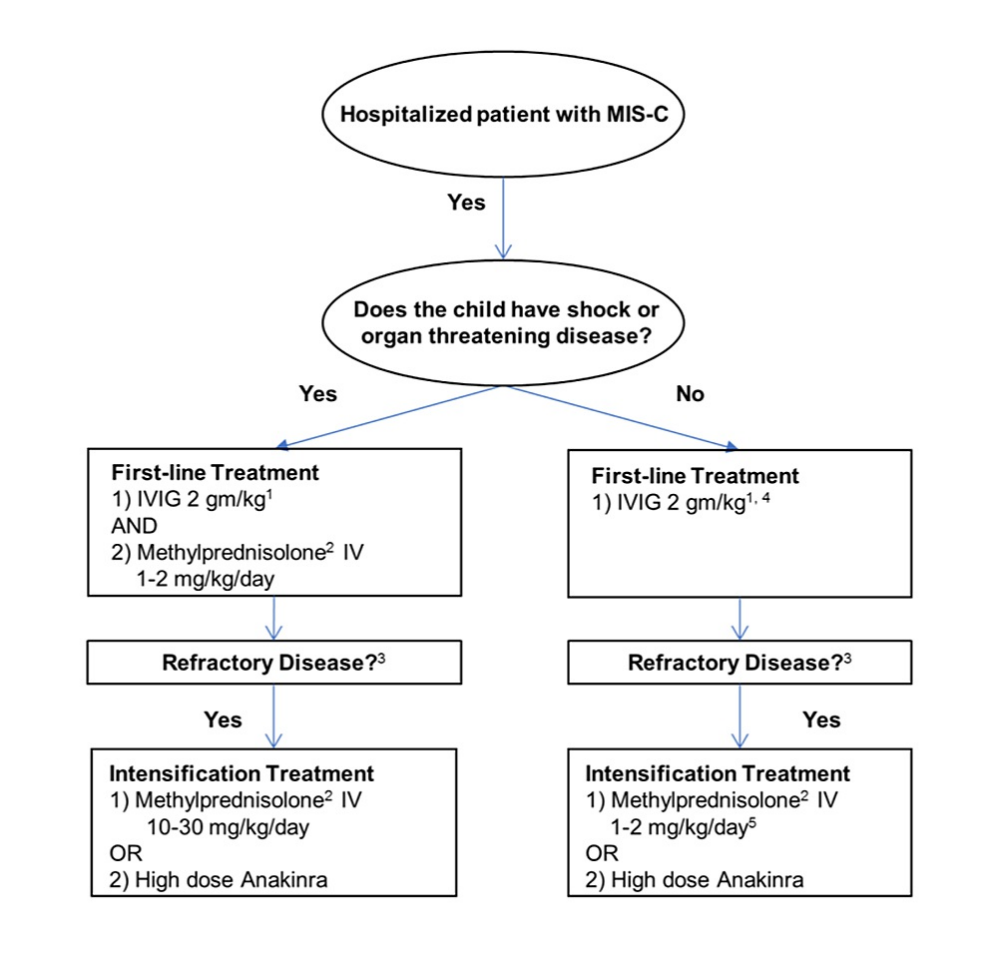
Algorithm for Initial Immunomodulatory Treatment in MIS-C MIS-C: multisystem inflammatory syndrome in children; IVIG: intravenous immunoglobulin.

The diagnostic criteria for MIS-C, presented by the American College of Rheumatology, defines an epidemiologic link to SARS-CoV-2 infection in a child with any of the following criteria: positive SARS-CoV-2 PCR, positive SARS-CoV-2 serologies, preceding illness resembling COVID-19, or close contact with confirmed or suspected COVID-19 cases in the past four weeks (Figure 1). Rash (polymorphic, maculopapular, or petechial, but not vesicular); GI symptoms (diarrhea, abdominal pain, or vomiting); oral mucosal changes (red and/or cracked lips, strawberry tongue, or erythema of the oropharyngeal mucosa); conjunctivitis (bilateral conjunctival injection without exudate); neurologic symptoms (altered metal status, encephalopathy, focal neurologic deficits, meningismus, or papilledema) [4]. Our approach outlined below is generally in line with already published guidance from the American College of Rheumatology and American Academy of Pediatrics (Figure 2). 

## Discussion

In April 2020, several cases were reported from London showing signs of acute inflammation and shock showing resemblances with KD or toxic shock syndrome. This was described as MIS-C by the Centers for Disease Control and Prevention (CDC). Since the initial reporting, multiple case clusters from all over the world have been reported and, as of October 1, 2020, there were 1,027 cases reported in the United States with a total of 20 deaths [13,14]. One case series described 2818 patients hospitalized with MIS-C criteria from February 2020 to March 2021 of whom 35 did not survive. 

A clinical criterion has been defined by the CDC since then for MIS-C [13,14]. We, in our case report, used the American College of Rheumatology criteria for the diagnosis of MIS-C which is almost like what CDC has suggested except that the age limit is <18 years [8]. Majority of children reported are Hispanic and Black (non-Hispanic) and have been predominantly in the 5-9 and 9-14 age groups while our case is of a 12-year-old of Arab descent.

The pathophysiology of MIS-C in children with COVID-19 is understood to be an infection triggering macrophage activation followed by helper T-cell activation. This later leads to massive cytokine release and the production of antibodies, which in turn results in immune dysregulation and a hyperimmune response. The presentation is of systemic signs of inflammation, often with significant cardiac, neurologic, and/or hematologic dysfunction. Our case has been fortunate to not develop a hyper-immune response and was detected at initial stages with only mild organ dysfunction. Most affected children have negative COVID-19 PCR but positive serology which confirms an epidemiologic link between COVID 19 and this illness. In most studies, a difference of several weeks was noted between the peak of COVID-19 cases within communities and the surge of MIS-C cases. The patient in this case report had positive COVID-19 PCR three weeks before the onset of symptoms and stayed positive till the time of diagnosis of MIS-C. We could not offer serological results as PCR was supporting the link.

Laboratory abnormalities in the vast majority of cases include variable white blood cell counts with predominantly neutrophilia (90%), high acute phase reactants (CRP -90 to 100%), ESR 75% to 80%, ferritin: 55% to 76%, and interleukin-6 (IL-6) - 80% to 100%. There is good evidence depicting elevated cardiac markers like troponin in 50% to 90% of cases along with hypoalbuminemia, mild increase in liver enzymes, and lactate dehydrogenase in 10% to 60%. The reported case showed most of the laboratory variations as reported in referenced studies. Cardiac involvement is not uncommon in MIS-C. In several large case series, about 30% to 40% of children had left ventricular dysfunction and 8% to 24% had coronary artery abnormalities [10]. Echocardiographic findings may include depressed LV function, coronary artery (CA) abnormalities, including dilation or aneurysm, mitral regurgitation, and pericardial effusion.

Outcomes of patients with MIS-C are difficult to interpret due to changing therapeutic strategies and geographical differences for available interventions. An international observational cohort study collected data regarding clinical outcomes. This data described the course of treatment for 614 children from 32 countries from June 2020 through February 2021. Among 614 children with suspected MIS-C, 246 received first-line therapy with IVIG alone, 208 with IVIG plus glucocorticoids, and 99 with glucocorticoids alone. Twenty-two children received second-line therapies including biologic agents, while 39 did not receive any immunomodulatory therapy. In this study, no difference was shown in recovery from MIS-C between various regimens of IVIG with and without glucocorticoids, or glucocorticoids alone. Various observational studies during the first, second, and third wave have shown that many SARS-CoV-2 variants had emerged. The reported patient also responded very well to first-line treatment with IVIG and had shown clinical improvement within 48 hours, as showed in other case reports and studies.

The prognosis of MIS-C looks good with a positive rate of recovery than mortality and morbidity. Results of long-term follow-up studies are expected over the course of time. Many children require intensive care facilities for their severe illnesses. A study of 46 children, admitted for MIS-C from April to September of 2020, who were later followed up in multidisciplinary clinics after discharge, showed common sequelae included muscular weakness, reduced exercise capacity, anxiety, and emotional difficulties [15].

Our patient was seen in the outpatient department after two weeks and he was in stable condition without any cardiac dysfunction on echocardiography and all his laboratory values were in the normal range.

## Conclusions

The incidence of MIS-C in persons younger than 21 years is very less and it is a rare complication of COVID-19. Our patient was the first case to be reported with this illness in the Qassim region of the Kingdom of Saudi Arabia. He had unremitting fever, generalized skin rash, conjunctivitis, congested oral mucosa with liver dysfunction, and high acute phase reactants to classify him as MIS-C based on the criteria by the American College of Rheumatology. He improved after IVIG and supportive management without any complications. The experience during the management of this case in understanding the clinical presentation helped us later in the recognition and treatment of two more cases with MIS-C. This case report and some help from the literature may assist other clinicians in identifying and managing such cases.
